# Cation dynamics by ^1^H and ^13^C MAS NMR in hybrid organic–inorganic (CH_3_CH_2_NH_3_)_2_CuCl_4_

**DOI:** 10.1039/c8ra06268d

**Published:** 2018-10-04

**Authors:** Ae Ran Lim, Yong Lak Joo

**Affiliations:** Analytical Laboratory of Advanced Ferroelectric Crystals, Jeonju University Jeonju 55069 South Korea aeranlim@hanmail.net arlim@jj.ac.kr +82-63-220-2514; Department of Science Education, Jeonju University Jeonju 55069 South Korea; School of Chemical and Biomolecular Engineering, Cornell University Ithaca, New York 14853 USA

## Abstract

To understand the dynamics of the cation in layered perovskite-type (CH_3_CH_2_NH_3_)_2_CuCl_4_, the temperature-dependent chemical shifts and spin–lattice relaxation times *T*_1ρ_ in the rotating frame have been measured using ^1^H magic angle spinning nuclear magnetic resonance (MAS NMR) and ^13^C cross-polarization (CP)/MAS NMR techniques. Each proton and carbon in the (CH_3_CH_2_NH_3_)^+^ cation is distinguished in MAS NMR spectra. The Bloembergen–Purcell–Pound (BPP) curves for ^1^H *T*_1ρ_ in CH_3_CH_2_ and NH_3_, and for the ^13^C *T*_1ρ_ in CH_3_ and CH_2_ are revealed to have minima at low temperatures. This implies that the curves represent the CH_3_ and NH_3_^+^ rotational motions. The amplitude of the cationic motion is enhanced at the C-end, that is, the N-end of the organic cation is fixed to the inorganic layer through N–H⋯Cl hydrogen bonds, and *T*_1ρ_ becomes short with larger-amplitude molecular motions.

## Abstract 2

To understand the dynamics of the cation in layered perovskite-type (CH_3_CH_2_NH_3_)_2_CuCl_4_, the temperature-dependent chemical shifts and spin–lattice relaxation times *T*_1ρ_ have been measured using ^1^H MAS NMR and ^13^C CP/MAS NMR techniques.

## Graphical abstract

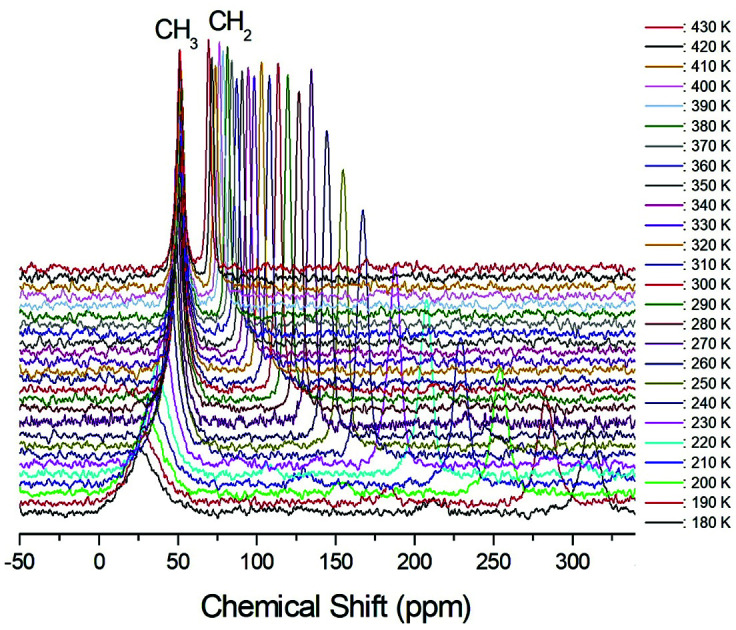

## I. Introduction

Metal–organic hybrids, which consist of organic and inorganic components, have recently attracted much attention because these materials have many possibilities for the tailoring of their functionalities and physical properties including optical, electrical and magnetic properties by adjusting the organic and/or metal building blocks. Hybrid metal–organic compounds based on the perovskite structures are of increasing interest due to their potential use for solar cells.^1,2^ However, toxicity and chemical instability issues of halide perovskites still remain as the main drawbacks for use in solar cells. The crystalline structure of compounds of the type (C_*n*_H_2*n*+1_NH_3_)_2_MCl_4_, where *n* = 1, 2, 3 … and M represents divalent metals (M = Cu, Cd, …), may be described as a sequence of alternating organic–inorganic layers.^3–6^ Many compounds in this family have been extensively investigated and have demonstrated successive phase transitions. This family of materials crystallizes in the layered perovskite structure, which consists of infinite, staggered layers of corner-sharing MCl_6_ octahedra interleaved by alkylammonium cations.^7^ Because of the layered character of their structure, these crystals become appropriate substances for investigations of two-dimensional electronic systems. The cavities between the octahedra are occupied by the ammonium heads of the organic cations, which, importantly, form strong N–H⋯Cl hydrogen bonds to any of the eight chloride ions.^8^

**Fig. 1:**
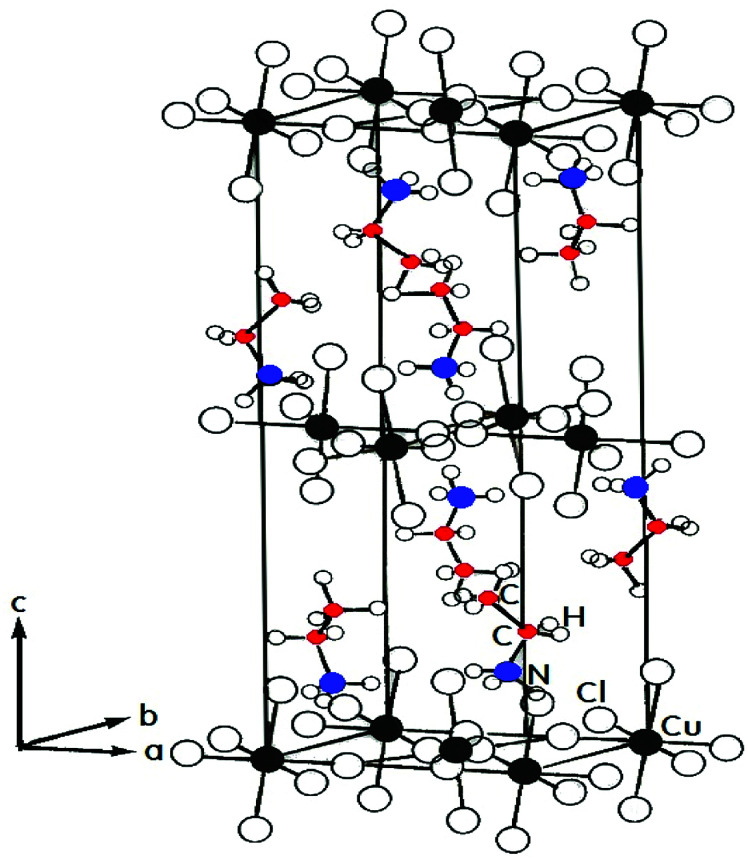
Orthorhombic structure of a (CH_3_CH_2_NH_3_)_2_CuCl_4_ crystal at room temperature.

Ethylammonium copper chloride (CH_3_CH_2_NH_3_)_2_CuCl_4_ is a layered perovskite-type compound that undergoes a complicated sequence of phase transitions. Differential scanning calorimetry (DSC) data indicates several phase transitions, at 236 K (=*T*_C4_), 330 K (=*T*_C3_), 357 K (=*T*_C2_), and 371 K (=*T*_C1_), as temperature increases.^9–14^ The peaks at 236 K, 330 K, and 371 K are very weak and can perhaps correspond to second-order transformations.^13^ The phase transitions in this crystal are mostly connected with changes in the arrangement of the alkylammonium chains. Fig. 1 shows the room-temperature orthorhombic crystal structure of (CH_3_CH_2_NH_3_)_2_CuCl_4_.^8,15^ The hybrids have the orthorhombic crystal structure with the space group *Pbca*, and the lattice constants are *a* = 7.47 Å, *b* = 7.35 Å, and *c* = 21.18 Å at room temperature.^16^ The CuCl_6_ octahedra are strongly distorted with elongated Cu–Cl bonds orthogonal to each other on adjacent octahedra. The CuCl_6_ sheets are sandwiched between two layers of alkylammonium. The structure of the organic component consists of a double layer of alkylammonium ions with their charged ends, the nitrogen atoms, oriented to the nearest CuCl_6_ plane.^4^ The complete structure is constituted by corner-sharing CuCl_6_ octahedra, forming the inorganic layers, and bilayers of organic cations attached to the octahedra by their NH_3_ heads.^17,18^

The structural geometry and molecular motions of the organic molecules within the layered hybrid structure is important for determining the influence of temperature on the evolution of the structural phase transitions in the perovskite structure. Physical properties in particular depend on the characteristics of metallic anion and the organic cation.

In the present study, the crystal structure and thermal stability for (CH_3_CH_2_NH_3_)_2_CuCl_4_ was observed by means of conventional X-ray, thermogravimetric analysis (TGA), and optical polarizing microscopy. In order to clarify the structural geometry and dynamics of the cation in the organic–inorganic (CH_3_CH_2_NH_3_)_2_CuCl_4_, we investigated the chemical shifts and the spin–lattice relaxation time *T*_1ρ_ in the rotating frame using ^1^H magic angle spinning nuclear magnetic resonance (MAS NMR) and ^13^C cross-polarization (CP)/MAS NMR. The CH_3_CH_2_ and NH_4_ groups of the CH_3_CH_2_NH_3_ cation are distinguishable in ^1^H MAS NMR spectra, and the CH_3_ and CH_2_ groups are distinguished by ^13^C CP/MAS NMR spectra. We investigated the ^1^H and ^13^C dynamics in the (CH_3_CH_2_NH_3_)^+^ cation near the phase-transition temperatures.

## II. Experimental method

Crystals of (CH_3_CH_2_NH_3_)_2_CuCl_4_ were obtained by slow evaporation at 25 °C from an aqueous solution of C_2_H_5_NH_2_·HCl and CuCl_2_·2H_2_O in the stoichiometric 2 : 1 proportion. The obtained crystals were yellow square plates, typically 5 mm × 5 mm in area and 0.5 mm in thickness.

The structure of the (CH_3_CH_2_NH_3_)_2_CuCl_4_ crystals was determined at room temperature with an X-ray diffraction system (PANalytical, X'pert pro MPD) with a Cu-Kα (*λ* = 1.5418) radiation source. Measurements were taken in a *θ*–2*θ* geometry from 10° to 60° at 45 kV and with a tube power of 40 mA. And, the TGA curve at a heating rate of 10 °C min^−1^ was measured under N_2_ atmosphere, and the mass of the powdered sample used in the TGA experiment was 11.41 mg.

The chemical shifts and the *T*_1ρ_ values for (CH_3_CH_2_NH_3_)_2_CuCl_4_ were obtained by ^1^H MAS NMR and ^13^C CP/MAS NMR at Larmor frequencies of *ω*_0_/2π = 400.13 and 100.61 MHz, respectively, using Bruker 400 MHz NMR spectrometers at the Korea Basic Science Institute, Western Seoul Center. Crystalline powdered samples were placed within a 4 mm CP/MAS probe, and the MAS rate for ^1^H and ^13^C measurements, to minimize spinning sideband overlap, was set to 10 kHz. The ^1^H *T*_1ρ_ values were determined using a π/2−*t* sequence by varying the duration of spin-locking pulses. ^13^C *T*_1ρ_ values were measured by varying the duration of the spin-locking pulse applied after the CP preparation period. The width of the π/2 pulse used for measuring *T*_1ρ_ for ^1^H and ^13^C was 3.7 μs, with the spin-locking field at 67.56 kHz. The chemical shifts and *T*_1ρ_ were measured over a temperature range of 180–430 K.

## III. Experimental results

The measured structure at room temperature exhibited orthorhombic symmetry with cell parameters of *a* = 7.480 Å, *b* = 7.375 Å, *c* = 21.254 Å for (CH_3_CH_2_NH_3_)_2_CuCl_4_ crystal. This result is consistent with the results reported by Steadman and Willett.^16^

**Fig. 2:**
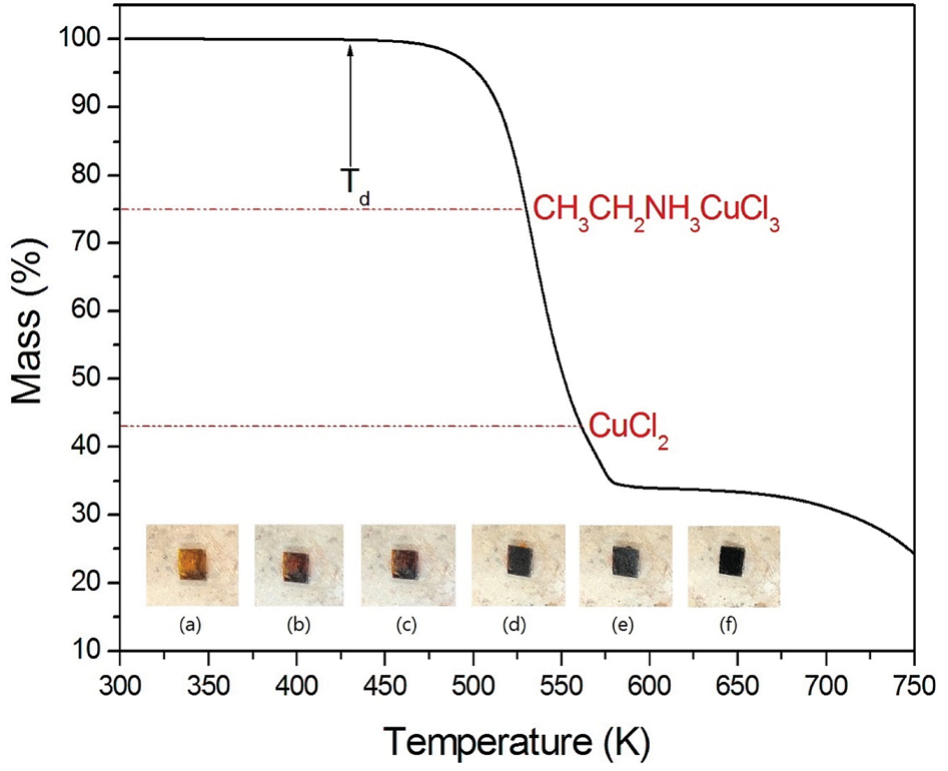
Thermogravimetric analysis of (CH_3_CH_2_NH_3_)_2_CuCl_4_ (inset: color changes of a (CH_3_CH_2_NH_3_)_2_CuCl_4_ crystal according to the temperature): (a) 300 K, (b) 350 K, (c) 400 K, (d) 450 K, (e) 500 K, and (f) 530 K.

The TGA curve of (CH_3_CH_2_NH_3_)_2_CuCl_4_ is shown in Fig. 2 for measuring thermal stability. The first occurrence of mass loss begins at approximately 430 K (*T*_d_), which is the onset of partial thermal decomposition. The second weight loss of 25.1% near 530 K is due to the removal of the CH_3_CH_2_NH_3_Cl from the compound, leaving intermediate CH_3_CH_2_NH_3_CuCl_3_ that belongs to another known class of compounds ABX_3_. Near 560 K, CuCl_2_ remains as the residue and when it reaches 580 K, the total weight loss becomes 65.55%. The color of the crystal is dark yellow at room temperature although it has slightly inhomogeneous hue due to surface roughness. As the temperature increases, the color of the crystal varies from dark yellow (300 K, 350 K), brown (400 K), to dark brown (450 K, 500 K), and then they start melting at 530 K as shown in the inset in Fig. 2. The TGA and optical polarizing microscopy results show that the crystal above 430 K allows CH_3_ to partially escape by the breaking the weak C–N bond.

**Fig. 3:**
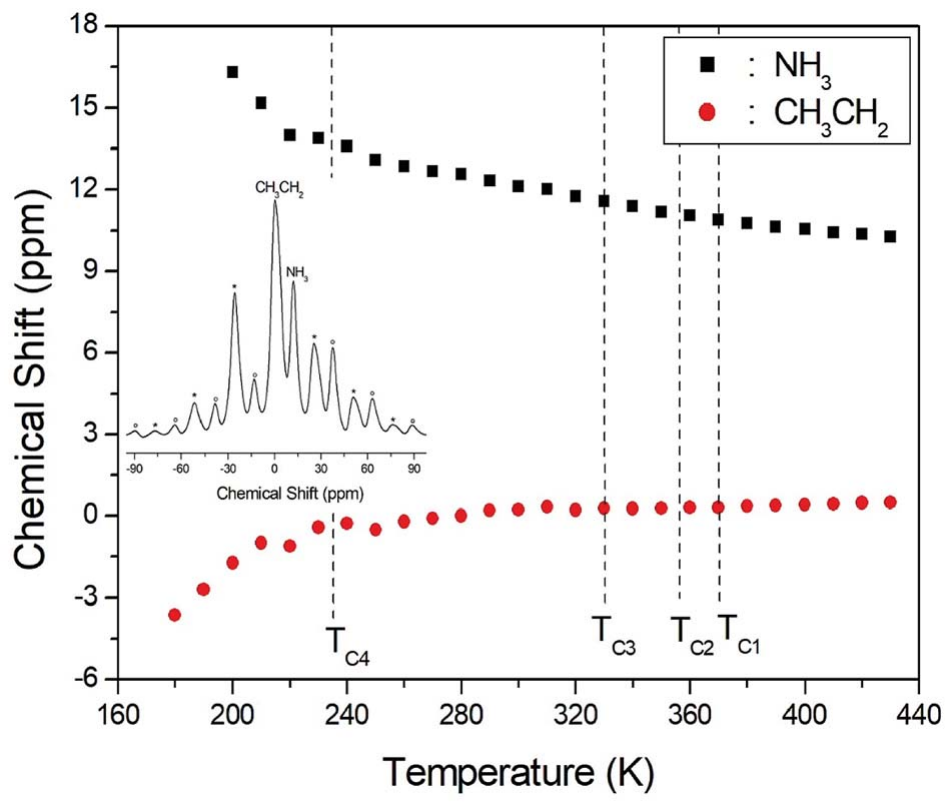
Chemical shifts for ^1^H MAS NMR of (CH_3_CH_2_NH_3_)_2_CuCl_4_ as a function of temperature (inset: ^1^H MAS NMR spectrum of (CH_3_CH_2_NH_3_)_2_CuCl_4_ at 300 K with spinning sidebands indicated by asterisks and open circles).

The ^1^H NMR spectra at a frequency of 400.13 MHz were obtained by MAS NMR. The ^1^H spectrum recorded at room temperature is shown in the inset in Fig. 3; the spectrum shows two peaks at chemical shifts of *δ* = 0.23 and 12.12 ppm, which are assigned to the protons of the CH_3_CH_2_ and NH_3_ groups, respectively. The spinning sidebands for CH_3_CH_2_ are marked with asterisks and those for NH_3_ are marked with open circles. However, the different ^1^H signals from CH_3_ and CH_2_ cannot be resolved, and therefore the combined CH_3_CH_2_ peak is very broad and has a larger intensity due to the overlap of the CH_3_ and CH_2_ peaks. The peak with the lower chemical shift is attributed to the protons in CH_3_CH_2_, and that of the higher chemical shift is attributed to the protons in NH_3_. The ^1^H chemical shifts for the alkyl and ammonium groups slowly and monotonously vary with temperature, indicating that the surrounding environments of the protons in the alkyl and ammonium groups change continuously, as shown in Fig. 3; here, the chemical shifts for protons in CH_3_CH_2_ and NH_3_ near *T*_C1_, *T*_C2_, and *T*_C3_ are nearly constant with temperature, whereas those for protons in CH_3_CH_2_ and NH_3_ below *T*_C4_ change more abruptly.

The *T*_1ρ_ values for the CH_3_CH_2_ and NH_3_ protons in (CH_3_CH_2_NH_3_)_2_CuCl_4_ were obtained as a function of temperature. The magnetization traces of both the alkyl and ammonium protons may be described by a single exponential function^19–21^

**Fig. 4:**
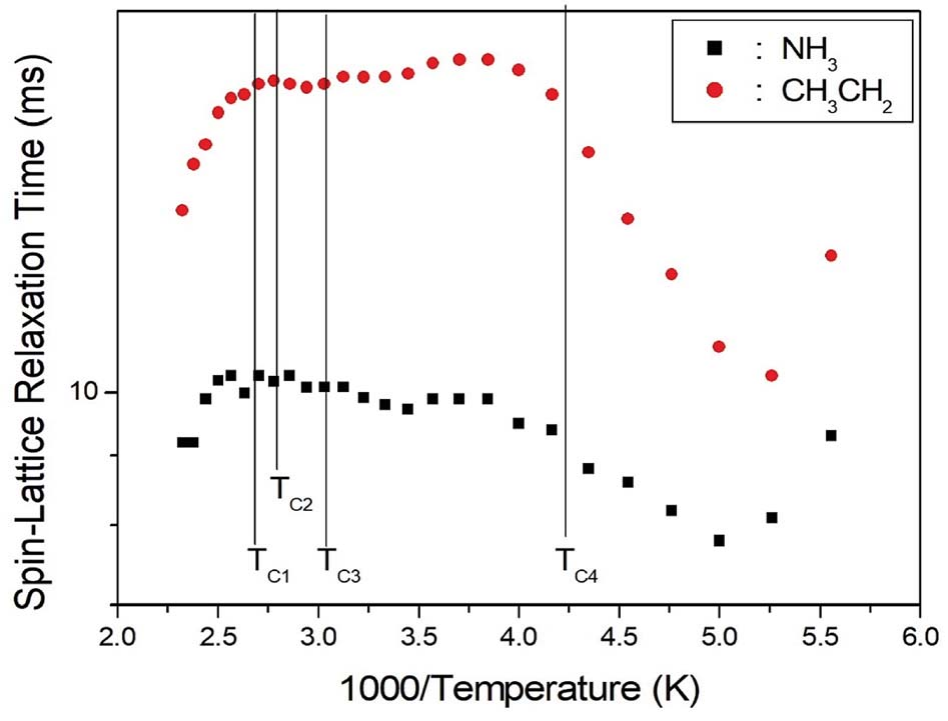
^1^H spin–lattice relaxation times *T*_1ρ_ in the rotating frame for the CH_3_CH_2_ and NH_3_ groups of (CH_3_CH_2_NH_3_)_2_CuCl_4_ as a function of inverse temperature.

1 *S*(*t*)/*S*_0_ = exp(−*t*/*T*_1ρ_),

where *S*(*t*) is the magnetization as a function of the spin-locking pulse duration *t*, and *S*_0_ is the total nuclear magnetization of the proton at thermal equilibrium.^19^ The recovery curves for several delay times were measured, and the *T*_1ρ_ values were obtained from the slopes by the delay time *vs.* intensity, at several different temperatures. This analysis method was used to obtain the *T*_1ρ_ values for each proton in CH_3_CH_2_ and NH_3_ which are plotted as a function of inverse temperature in Fig. 4. The *T*_1ρ_ values for the CH_3_CH_2_ and NH_3_ protons in the (CH_3_CH_2_NH_3_)^+^ cations exhibit similar trends with temperature. The proton *T*_1ρ_ data do not show evidence of a change near the phase-transition temperature; the *T*_1ρ_ values of protons in the CH_3_CH_2_ and NH_3_ groups of (CH_3_CH_2_NH_3_)_2_CuCl_4_ are almost continuous near *T*_C1_, *T*_C2_, and *T*_C3_, and these values are of the order of few milliseconds. The *T*_1ρ_ values abruptly decreased with temperature in the region approaching *T*_C4_. The relaxation time for the ^1^H nucleus is minimal at 190 K and 200 K for CH_3_CH_2_ and NH_3_, respectively. This feature of *T*_1ρ_ indicates that distinct molecular motions are present. The *T*_1ρ_ values are related to the corresponding values of the rotational correlation time, *τ*_C_, which is a direct measure of the rate of molecular motion. For the spin–lattice relaxation time in the rotating frame, the experimental value of *T*_1ρ_ can be expressed in terms of the correlation time *τ*_C_ for the molecular motion, as suggested by the Bloembergen–Purcell–Pound (BPP) function:^19,22^

2 *T*_1ρ_^−1^ = (*N*/20)(*γ*_H_*γ*_C_*ħ*/*r*_H–C_^3^)^2^{4*τ*_C_/(1 + *ω*_1_^2^*τ*_C_^2^) + *τ*_C_/[1 + (*ω*_H_ − *ω*_C_)^2^*τ*_C_^2^] + 3*τ*_C_/[1 + (*ω*_C_^2^*τ*_C_^2^)] + 6*τ*_C_/[1 + (*ω*_H_ + *ω*_C_)^2^*τ*_C_^2^] + 6*τ*_C_/[1 + *ω*_H_^2^*τ*_C_^2^]}.

**Fig. 5:**
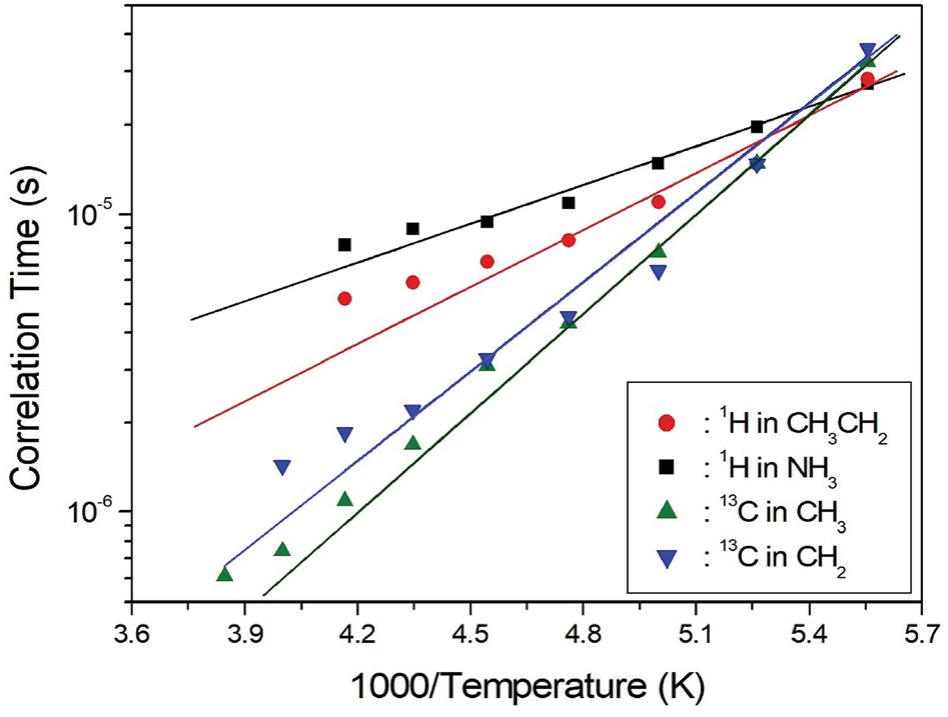
Arrhenius plots of the natural logarithm of the correlation time for each ^1^H and ^13^C of (CH_3_CH_2_NH_3_)_2_CuCl_4_ as a function of inverse temperature.

Here, *γ*_H_ and *γ*_C_ are the gyromagnetic ratios for the ^1^H and ^13^C nuclei, respectively; *N* is the number of directly bound protons; *r*_H–C_ is the H–C internuclear distance; *ħ* is the reduced Planck constant; *ω*_H_ and *ω*_C_ are the Larmor frequencies of ^1^H and ^13^C, respectively; and *ω*_1_ is the frequency of the spin-locking field. We analyzed our data assuming that *T*_1ρ_ would show a minimum when *ω*_1_*τ*_C_ = 1, and that the BPP relation between *T*_1ρ_ and the characteristic frequency *ω*_1_ could be applied. We sensitively controlled the minima in the *T*_1ρ_ temperature variations and the slopes around the minima. From these results, the value of (*γ*_H_*γ*_C_*ħ*/*r*_H–C_^3^)^2^ for the proton in eqn (2) was obtained. We then calculated the temperature dependences of the *τ*_C_ values for protons by using the obtained values of (*γ*_H_*γ*_C_*ħ*/*r*_H–C_^3^)^2^. The temperature dependence of *τ*_C_ follows a simple Arrhenius equation:

3 *τ*_C_ = *τ*_0_ exp(−*E*_a_/*RT*),

where *τ*_0_ is a pre-exponential factor, *T* is the temperature, *R* is the gas constant, and *E*_a_ is the activation energy. Thus, the slope of the linear portion of a semi-log plot should yield *E*_a_. The *E*_a_ value for the rotational motion can be obtained from the log *τ*_C_*vs.* 1000/*T* curve shown in Fig. 5; we obtained *E*_a_ = 12.19 ± 1.30 kJ mol^−1^ and *E*_a_ = 8.33 ± 0.50 kJ mol^−1^ for CH_3_CH_2_ and NH_3_, respectively. The rotational motion for alkyl groups is activated, whereas the rotational motion for ammonium groups at the end of the organic cation is less strongly activated.

**Fig. 6:**
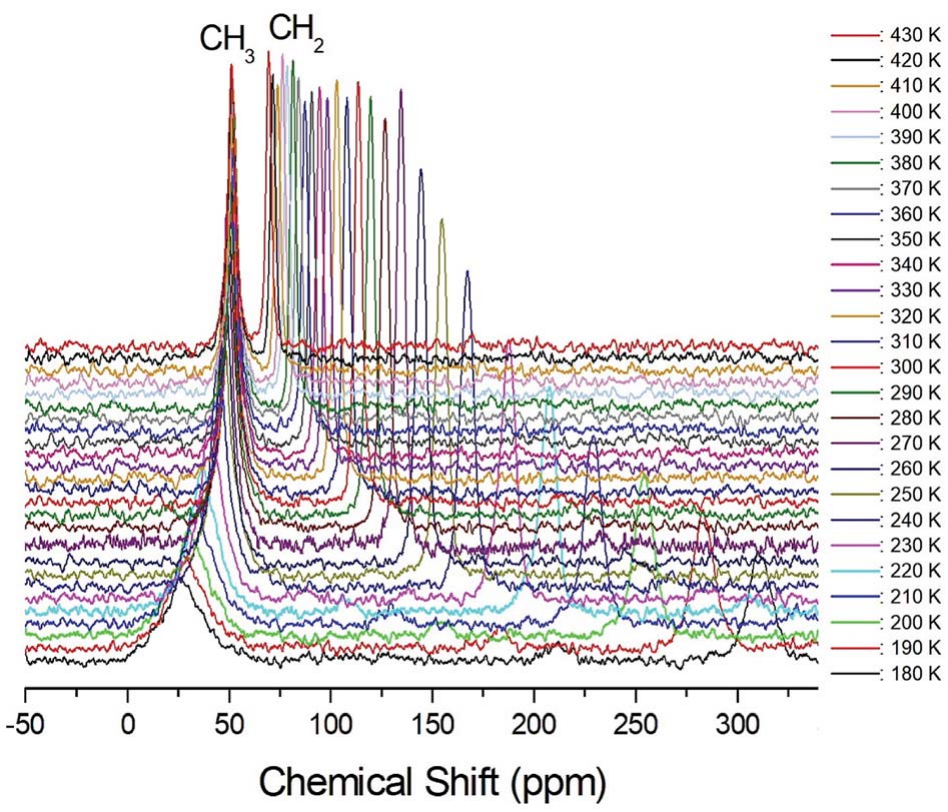
^13^C CP/MAS NMR spectra of (CH_3_CH_2_NH_3_)_2_CuCl_4_ measured at different temperatures.

**Fig. 7:**
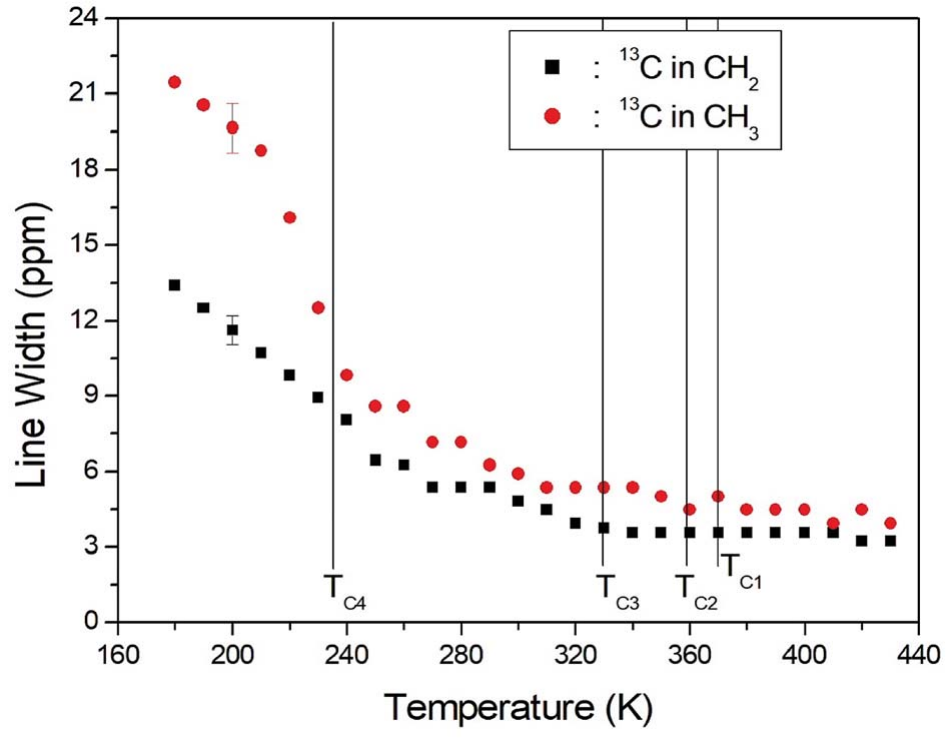
Temperature dependences of line widths of ^13^C NMR spectra of CH_3_ and CH_2_ in (CH_3_CH_2_NH_3_)_2_CuCl_4_.

The structural analysis of the carbons in (CH_3_CH_2_NH_3_)_2_CuCl_4_ was performed by ^13^C CP/MAS NMR, and the corresponding spectrum is shown in Fig. 6, as a function of temperature; the ^13^C CP/MAS NMR spectrum at room temperature shows two signals at chemical shifts of *δ* = 50.77 ppm and *δ* = 113.50 ppm with respect to tetramethysilane (TMS), which can be assigned to CH_3_ and CH_2_, respectively. The ^13^C chemical shift of CH_2_ abruptly shifts with temperature, whereas that of CH_3_ changes only much less with temperature. The full width at half maximum (FWHM) linewidths for the ^13^C of CH_3_ and CH_2_ in Fig. 7 showed a monotonic decrease with increasing temperature, with no particular anomalies attributable to the phase transitions. The linewidth of the ^13^C signal assigned to CH_3_ is broad compared to that of CH_2_, and the linewidth narrows significantly with increasing temperature. This narrowing of the ^13^C linewidths is attributed to internal motions that the line widths follow the same temperature dependence as some internal motions, hence the motions are responsible for the line widths.

**Fig. 8:**
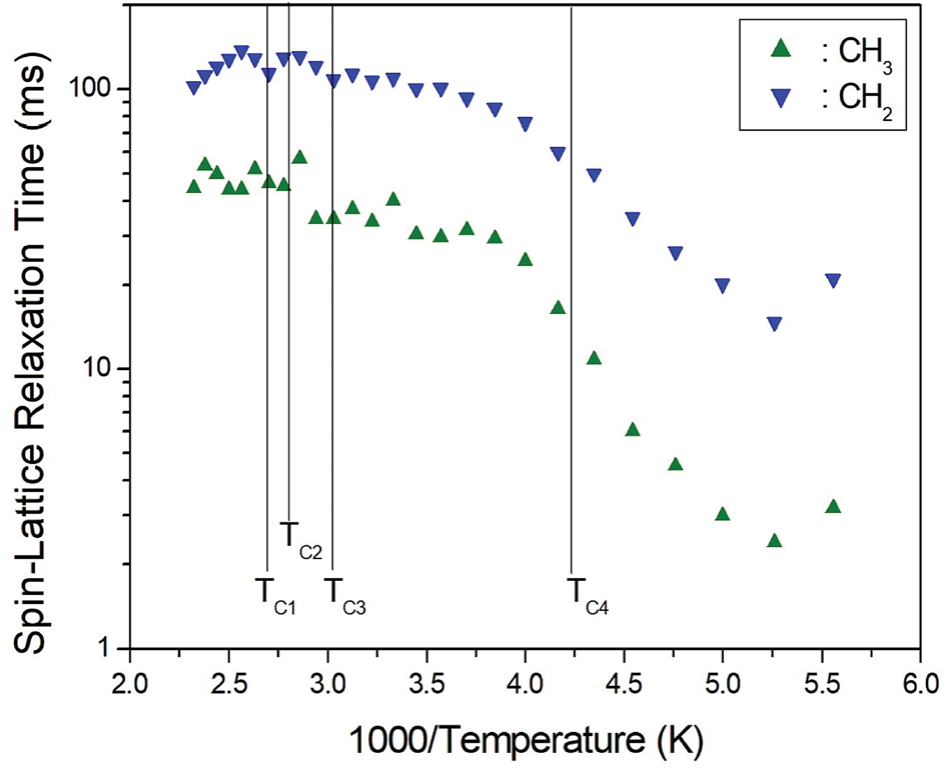
^13^C spin–lattice relaxation times *T*_1ρ_ in the rotating frame for CH_3_ and CH_2_ in (CH_3_CH_2_NH_3_)_2_CuCl_4_ as a function of inverse temperature.

To obtain the ^13^C *T*_1ρ_ values, the nuclear magnetization was also measured at several temperatures as a function of delay time. The signal intensity of the nuclear magnetization recovery curves for ^13^C is described by a single exponential function as in eqn (1) at all temperatures. The ^13^C *T*_1ρ_ values for CH_3_ and CH_2_ in (CH_3_CH_2_NH_3_)_2_CuCl_4_ are plotted as a function of inverse temperature in Fig. 8. The temperature dependences of the ^13^C MAS NMR *T*_1ρ_ values seem to be similar. The *T*_1ρ_ values for CH_3_ and CH_2_ both increase with temperature in the same manner; whereas, the ^13^C *T*_1ρ_ values near the phase-transition temperatures are approximately continuous. The *T*_1ρ_ values for CH_3_ and CH_2_ at room temperature are 33.85 ms and 109.40 ms, respectively. The amplitude of the cationic motion is enhanced at its CH_3_ end, and the central CH_2_ moiety is fixed to the NH_3_ group in the organic cation. The *T*_1ρ_ curve below *T*_C4_ can be reproduced by BPP theory. The BPP curves for CH_3_ and CH_2_, showing minima at low temperatures, is almost the same as those of the CH_3_CH_2_ and NH_3_ shifts of the ^1^H MAS NMR measurements. *E*_a_ for the rotational motion of CH_3_ and CH_2_ can be obtained from the log *τ*_C_*vs.* 1000/*T* curve shown in Fig. 5; we obtained *E*_a_ = 21.35 ± 0.45 kJ mol^−1^ for CH_3_ and *E*_a_ = 19.72 ± 1.76 kJ mol^−1^ for CH_2_, respectively, which, considering their error ranges, are the same values.

## IV. Conclusion

We discuss the molecular motions for cation of Cu-based hybrid materials, where we replace Pb with nontoxic Cu metal for lead-free perovskite solar cells, and investigate their potential toward solar cell applications based on ionic dynamics of the cation in hybrid organic–inorganic (CH_3_CH_2_NH_3_)_2_CuCl_4_ by NMR studies. The cation dynamics and interionic interactions through hydrogen bonds are expected to be closely related with the physical properties due to the potential applications. The cation dynamics in a layered perovskite-type (CH_3_CH_2_NH_3_)_2_CuCl_4_ were investigated as a function of temperature by ^1^H MAS NMR and ^13^C CP/MAS NMR experiments. The CH_3_CH_2_ and NH_4_ units in the CH_3_CH_2_NH_3_ cation were distinguished by the ^1^H MAS NMR spectra, and the CH_3_ and CH_2_ units in the CH_3_CH_2_NH_3_ cation were also clearly distinguished in the ^13^C CP/MAS NMR spectra. To obtain detailed information about the cation dynamics of this crystal, the spin–lattice relaxation time *T*_1ρ_ in the rotating frame for both ^1^H and ^13^C were measured, revealing that these atoms undergo rotational motions at low temperatures. The BPP curves for the ^1^H *T*_1ρ_ in CH_3_CH_2_ and NH_3_, and for the ^13^C *T*_1ρ_ in CH_3_ and CH_2_, were shown to have a minimum at low temperatures; the *T*_1ρ_ of ^1^H and ^13^C showed a minimum and is governed by the tumbling motion of the CH_3_CH_2_ and NH_3_ groups, indicating that the ^1^H and ^13^C atoms in the CH_3_CH_2_NH_3_^+^ groups exhibit high mobility at low temperatures. The molecular motions for ^1^H and ^13^C in the CH_3_CH_2_NH_3_^+^ cation were very free at low temperatures. *T*_1ρ_ provides insight into the changes in the cation reorientation rates at low temperature.

The ^13^C *T*_1ρ_ values in CH_3_ increased with temperature, a trend that has been observed in alkyl chains attached to the (CH_3_CH_2_NH_3_) cation due to its greater mobility toward its free end. The CH_3_CH_2_NH_3_ cationic motion is enhanced at the opposing end of the cation to the NH_4_^+^ group probably because this group is bound to the inorganic layer through the N–H⋯Cl hydrogen bonds. The ^13^C *T*_1ρ_ is usually dominated by the fluctuation of the anisotropic chemical shift, and it becomes shorter with larger-amplitude molecular motions. This implies that the amplitude of the cationic motion is enhanced at the C-end, that is, the N-end of the organic cation is fixed at the inorganic layer through N–H⋯Cl hydrogen bonds. The cationic motion, being associated with the fluctuation of the molecular axis, is expected to be gradually excited with increasing temperature.

## Conflicts of interest

There are no conflicts to declare.

## Supplementary Material
